# A Rare Case of Hemorrhagic Shock: Morel-Lavallée Lesion

**DOI:** 10.5811/cpcem.2019.9.43674

**Published:** 2019-10-21

**Authors:** Lieke Claassen, Myriam Anna Franssen, Erik Robert de Loos

**Affiliations:** * Zuyderland Medical Centre, Department of Emergency Medicine, Heerlen, The Netherlands; † Zuyderland Medical Centre, Department of Surgery, Heerlen, The Netherlands

## Abstract

Hemorrhage is a major cause of death among trauma patients. Controlling the bleeding is essential but can be difficult when the source of bleeding remains unidentified. We present a 67-year-old healthy male with a hypovolemic shock after a suicide attempt by jumping from a height. Apart from a bilateral pneumothorax with multiple rib fractures, a femur fracture and spine fractures, computer tomography (CT) revealed a closed, degloving injury of the back, also known as a Morel-Lavallée lesion. Hemodynamic instability due to hemorrhage caused by a Morel-Lavallée lesion in the lumbar region is very rare and easily overlooked. This case demonstrates the importance of clinical signs of Morel-Lavallée, and illustrates the need for total body CTs to exclude other locations of bleeding and to detect contrast extravasation. This report also discusses the possible treatment options for Morel-Lavallée lesions.

## INTRODUCTION

Hemorrhage is the most common cause of shock in trauma patients and a major cause of death among them.^1^ Failure to provide the correct treatment to control the bleeding contributes to preventable trauma death. A structural and uniform initial assessment according to Advanced Trauma Life Support (ATLS) guidelines is essential for early identification of bleeding and hemostasis. A timely log roll could be warranted, especially when the focus of hemorrhage is unclear. Massive soft tissue injury is an underestimated cause of hypovolemic shock. We present a trauma patient with a rare case of hemorrhagic shock due to a Morel-Lavallée lesion in the lumbar region. We also discuss the management of this injury.

## CASE REPORT

A 67-year-old male, with no known health issues prior to presentation at the emergency department (ED), arrived at the ED via ambulance after a suicide attempt by jumping from a height. The patient was assessed according to the ATLS guidelines. On arrival, his breathing was spontaneous and he was able to speak.

The patient had the following vital signs: respiratory rate of 18 breaths per minute, 100% oxygen saturation on 15-liter (L) non-rebreathing mask, heart rate of 108 beats per minute, blood pressure: 86/40 millimeters of mercury, and a temperature of 36.5° Celsius. The Glasgow Coma Scale (GCS) was 14 (motor: four, verbal: six, eyes: four) and pupils were symmetric and reactive to light. On physical examination his lungs were bilaterally clear to auscultation. The abdomen was soft with no abdominal tenderness and the pelvis was clinically stable. The left femur appeared shortened and rotated. His neurological exam did not demonstrate any localized neurological deficit. Motor strength was 5/5 in the upper extremities and -/5 in the lower extremities due to a femur fracture. Sensory testing was normal and reflexes were symmetrical low with a normal Babinski reflex.

A back examination revealed a large, boggy swelling on the lower back. The patient received two L of warmed saline and four units of packed red blood cells after which the circulatory status normalized. Additional radiological imaging was performed. Chest radiography showed multiple rib fractures. A focused assessment sonography for trauma (FAST) showed no free intraperitoneal fluid. No pericardial effusion was detected.

**Image 1:**
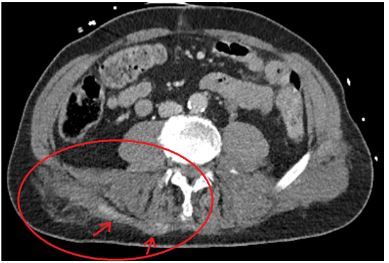
Computed tomography scan of the abdomen on arrival at the emergency department showing the early signs of a Morel-Lavallée lesion in the lumbar region. The oval shows the main lesion. The arrows indicate the contrast extravasation.

As we did not yet have a clear explanation for the ongoing shock, computer tomography (CT) was performed. CT imaging included the head, cervical-, thoracic- and lumbar spines, chest, abdomen, pelvis and femora. This CT showed unstable fractures of the first to the fourth lumbar vertebrae, bilateral multiple rib fractures with a small bilateral pneumothorax, a left proximal femur fracture, and an extensive Morel-Lavallée lesion in the lumbar region with minor contrast extravasation (Image 1). There were no signs of a spinal cord lesion.

The patient was transferred to the intensive care unit for further resuscitation. Temporary skeletal traction was applied for the proximal femoral fracture. Conservative treatment of the Morel-Lavallée lesion was initiated, allowing for spontaneous tamponade of the bleeding due to compression from the patient’s own body weight. In-line spinal immobilization was maintained.

**Image 2:**
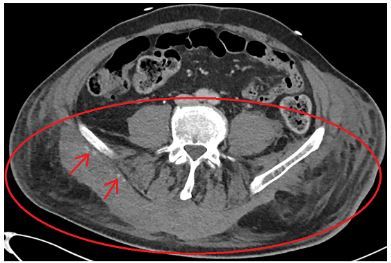
Computed tomography of the abdomen eight hours after admission with a Morel-Lavallée lesion in the lumbar region. A mass is visible on the dorsal side of the pelvis. The oval shows the main lesion. The arrows indicate the contrast extravasation.

A CT angiography was repeated eight hours after admission because of an ongoing need for inotropic support. A persistent, active bleeding in the Morel-Lavallée lesion was observed (Image 2). Because of the accompanying unstable fractures of the lumbar vertebrae, surgical treatment or coiling was deemed high risk, and therefore conservative treatment was continued. During the following 24 hours the hemodynamic status normalized and inotropic support was discontinued. After one week, a successful spondylodesis of the lumbar spine as well as an osteosynthesis of the proximal femur fracture was performed. The patient recovered well without any complications. He was discharged to a rehabilitation center on day 64.

## DISCUSSION

A Morel-Lavallée lesion is a closed, degloving injury first described by the French surgeon Victor Auguste Francois in 1863.^2^ It is also known as a post-traumatic, soft tissue cyst or a chronic expanding hematoma. In most cases, this lesion is the result of severe injury due to high-energy trauma. It is also reported in contact sports and in postoperative complications.^3^,^4^ Severe shearing forces during trauma cause separation between the loose skin, subcutaneous fat, and the relatively immobile underlying deep fascia. This results in hemolymphatic fluid collection originating from the disrupted blood vessels and lymphatic vessels. Morel-Lavallée lesions can involve only soft tissue or they can occur in combination with fractures. Apart from local pain and a soft boggy swelling, hypoesthesia in the affected region is common due to damage to cutaneous nerve branches.^2^,^3^

**CPC-EM Capsule**

What do we already know about this clinical entity?

*A Morel-Lavallée lesion can occur in the lumbar region but hemodynamic instability is rare. There are different treatment modalities such as surgical debridement or percutaneous drainage*.

What makes this presentation of disease reportable?

*In this case the Morel-Lavallée lesion in the lumbar region caused significant hemodynamic instability and the total body computed tomography scan showed significant contrast extravasation*.

What is the major learning point?

*A Morel-Lavallée lesion can cause significant hemodynamic instability and a full body examination is important. Pressure of the patients own body weight can be a sufficient treatment*.

How might this improve emergency medicine practice?

*Compression of the patients own body weight can be a considered treatment in patients with a Morel-Lavallée lesion in the lumbar region to regain hemodynamic stability*.

Little is known about the epidemiology of Morel-Lavallée lesions. Vanhegan et al. reviewed the location of 204 Morel-Lavallée lesions in 29 papers. The lesions are most commonly found at the following locations: greater trochanter or hip–30.4%; thigh–20.1%; pelvis–18.6%; knee–15.7%; gluteal–6.4%; lumbosacral–3.4%; abdominal wall–1.5%; calf or lower leg–1.5%; head–0.5%; and unspecified–2.0%. Only a few published case reports describe a Morel-Lavallée lesion in the lumbar region, and they are mainly chronic lesions in non-shock patients.^3^ Only three cases of hemorrhagic shock have been described.^5^,^6^,^7^

Morel-Lavallée lesions often evolve over a few hours to days after an injury. However, up to 30% of Morel-Lavallée lesions develop over months following the initial injury, of which 30% remain undiagnosed.^8^,^9^ These lesions are often missed or mistaken for tumors (sarcoma), soft tissue hematomas, fat necrosis, pseudo-lipoma, abscesses or a bursitis. This often causes a delay in treatment.

Several diagnostic imaging modalities can help to diagnose a Morel-Lavallée lesion. These include CT and magnetic resonance imaging (MRI).^9^,^10^ Ultrasound has also proved to be effective for diagnosing, monitoring and follow-up, but due to the stages of internal blood product degeneration (seroma, subacute hematoma, and chronic organized hematoma), their presentation can vary over time and these lesions can be difficult to detect.^11^

A CT is usually easier to perform in the acute situation. Early lesions typically demonstrate the CT characteristics associated with hematomas. Only a third of the lesions show active contrast extravasation at the time of the initial scan. McKenzie et al. stated that the possibility of a closed, soft tissue degloving injury should be raised in the clinical setting of a high-energy trauma in combination with a fluid collection in the subcutaneous tissues overlying the deep fascia, with sparing of the overlying skin and internal fat globules on CTs. The clinical setting is highly important while examining the CT, otherwise a Morel-Lavallée lesion can often go unrecognized.^12^ Despite this recent research by McKenzie et al., who considered CT imaging to be a reliable detection method, MRI is still the gold standard in diagnosing Morel-Lavallée lesion at its different stages.^10^ In its early stages, the hemolymphatic fluid appears hyperintense on T2 images. At a later stage, hemoglobin in the hemolymphatic fluid transforms to methemoglobin, which causes an increased intensity on T1 images. In the final stage, a surrounding capsule develops that will be visible on MRI images.^13^, ^14^

Traditionally, surgical open debridement has been the preferred treatment of a Morel-Lavallée lesion. Over time, less invasive methods with better aesthetic outcomes have been developed, including (imaging-guided) percutaneous drainage, compressive bandaging and injection of sclerosing agents such as doxycycline, fibrin glue, and alcohol.^15^,^16^ Sclerodesis is the favored method in patients without fractures. When the lesion is older and encapsulated, surgical excision is more commonly used.

Even though a Morel-Lavallée lesion is a closed injury, potential complications include soft tissue or deep bone infection, wound dehiscence, and skin necrosis. Separation of the vasculature due to the shearing mechanism can result in skin necrosis. This may also be a result of the mass effect of the fluid collection, which further compresses the supplying vascular plexus, causing pressure-related ischemia.^8^

To date, only three cases of a patient in shock due to a Morel-Lavallée lesion have been reported. Mao et al. reported a lesion located at the upper thigh, which was treated by surgical drainage after a few days.^7^ Hefny et al. reported a case of an extensive hematoma at the flank without contrast extravasation on the CT.^5^ In this case, percutaneous suction drainage was performed. Yumoto et al. reported a lesion of the lower back. An initial CT detected contrast extravasation, and transcatheter arterial embolization was successfully performed.^6^

In our case the Morel-Lavallée lesion was situated at an uncommon location. The lesion developed in a very short time and it caused hemodynamic instability. Per ATLS guidelines there was, at minimum, class III shock based on tachycardia, hypotension, and confusion.^17^ In trauma patients, the most likely cause of shock is bleeding unless there is an obvious alternative cause. In addition to the swelling on the back, the patient had a femur fracture, and multiple rib and spinal fractures, which could also have caused bleeding. In light of the fact that we applied traction on the femur, the ongoing shock was, in our opinion, the result of the Morel-Lavallée lesion on the patient’s back. There was no sign of cardiogenic shock or cardiac tamponade and there was no tension pneumothorax. Although only three cases have been reported, Morel-Lavallée lesions can contribute to or be responsible for hemorrhagic shock in trauma.

The traditional screening in trauma patients involving chest and pelvic radiographs and FAST cannot detect all bleeding sites. Physicians should check for occult bleeding in patients with hemorrhagic shock with an unknown focus. In hemodynamically unstable patients, contrast-enhanced CT may be needed to find the source of bleeding. However, particularly in patients with clinical signs of a Morel-Lavallée lesion, a contrast-enhanced CT is advised to exclude other foci of bleeding. In our case, the vertebral fractures made it difficult to use one of the recommended treatments as described above. In the two similar cases of Hefny et al. and Yumoto et al. they chose to drain the lesion. We believe that external compression is a suitable alternative treatment, especially if the lesion is located in the lumbar region and accompanied by unstable fractures. Compression can be achieved by using the patient’s own body weight. This seems to be a practical approach that is not always considered.

## CONCLUSION

Hemodynamic instability due to hemorrhage caused by a Morel-Lavallée lesion in the lumbar region is very rare and easily overlooked. In hemodynamically unstable patients a contrast-enhanced CT is advised to detect the bleeding focus. When a Morel-Lavallée lesion is situated in the lumbar region, conservative treatment by compression using the patient’s own body weight can be an appropriate therapy.
